# Development of in House ELISAs to Detect Antibodies to SARS-CoV-2 in Infected and Vaccinated Humans by Using Recombinant S, S1 and RBD Proteins

**DOI:** 10.3390/diagnostics12123085

**Published:** 2022-12-07

**Authors:** Aysun Yilmaz, Nuri Turan, Bekir Sami Kocazeybek, Harika Oyku Dinc, Hasan Emre Tali, Ozge Aydin, Hamid Besim Tali, Semaha Gul Yilmaz, Dildar Konukoglu, Sermin Borekci, Dashzeveg Bold, Gleyder Roman Sosa, Nejdiye Gungordu, Ilgim Vardaloglu, Nesrin Gareayaghi, Mine Guzel, Ebru Guner, Jean-Remy Sadeyen, Pengxiang Chang, Munir Iqbal, Juergen A. Richt, Huseyin Yilmaz

**Affiliations:** 1 Department of Virology, Veterinary Faculty, Istanbul University-Cerrahpasa, Hadimkoy, Istanbul 34098, Turkey; 2 The Pirbright Institute, Ash Road, Pirbright, Woking GU24 0NF, UK; 3 Department of Medical Microbiology, Cerrahpasa Faculty of Medicine, Istanbul University-Cerrahpasa, Istanbul 34098, Turkey; 4 Department of Pharmaceutical Microbiology, Faculty of Pharmacy, Bezmialem Vakif University, Istanbul 34098, Turkey; 5 Department of Biocehmistry, Cerrahpasa Faculty of Medicine, Istanbul University-Cerrahpasa, Istanbul 34098, Turkey; 6 Department of Pulmonary Diseases, Cerrahpasa Faculty of Medicine, Istanbul University-Cerrahpasa, Istanbul 34098, Turkey; 7 Department of Diagnostic Medicine and Pathobiology, College of Veterinary Medicine, Kansas State University, Manhattan, KS 66506, USA; 8 Department of Occupational Diseases, Cerrahpasa Faculty of Medicine, Istanbul University-Cerrahpasa, Istanbul 34098, Turkey; 9 Sisli, Hamidiye Etfal Training and Research Hospiatal, Blood Center, Istanbul 34098, Turkey; 10 Biruni Laboratories, Esentepe, Istanbul 34098, Turkey

**Keywords:** SARS-CoV-2, recombinant, S, S1, RBD, ELISA, human, Turkey

## Abstract

(1) Background: The aim of this study was to produce in-house ELISAs which can be used to determine SARS-CoV-2-specific antibody levels directed against the spike protein (S), the S1 subunit of S and the receptor binding domain (RBD) of S in SARS-CoV-2 vaccinated and infected humans. (2) Methods: Three in-house ELISAs were developed by using recombinant proteins of SARS-CoV-2, namely the S, S1 and RBD proteins. Specificity and sensitivity evaluations of these tests were performed using sera from SARS-CoV-2-infected (*n* = 70) and SARS-CoV-2-vaccinated (*n* = 222; CoronaVac vaccine) humans in Istanbul, Turkey. The analyses for the presence of SARS-CoV-2-specific antibodies were performed using the in-house ELISAs, a commercial ELISA (Abbott) and a commercial surrogate virus neutralization test (sVNT). We also analyzed archival human sera (*n* = 50) collected before the emergence of COVID-19 cases in Turkey. (3) Results: The sensitivity of the in-house S, S1 and RBD ELISAs was found to be 88.44, 90.17 and 95.38%, while the specificity was 72.27, 89.08 and 89.92%, respectively, when compared to the commercial SARS-CoV-2 antibody test kit. The area under curve (AUC) values were 0.777 for the in-house S ELISA, 0.926 for the S1 ELISA, and 0.959 for the RBD ELISA. The kappa values were 0.62, 0.79 and 0.86 for the S, S1 and RBD ELISAs, respectively. (4) Conclusions: The in-house S1 and RBD ELISAs developed in this study have acceptable performance characteristics in terms of sensitivity, specificity, AUC and kappa values. In particular, the RBD ELISA seems viable to determine SARS-CoV-2-specific antibody levels, both in infected and vaccinated people, and help mitigate SARS-CoV-2 outbreaks and spread.

## 1. Introduction

Severe respiratory infections in humans were first reported in December 2019 in Wuhan, China; the etiological agent was later characterized as severe acute respiratory syndrome coronavirus 2 (SARS-CoV-2), and the disease was designated as coronavirus disease 2019 (COVID-19) by the World Health Organization (WHO) [1]. COVID-19 became a pandemic in spring of 2020 and has been causing very serious threats to public health and economies globally [2,3]. Coronaviruses (CoVs) are enveloped, single-stranded RNA viruses belonging to the *Coronaviridae* family; they include a number of zoonotic viruses and are classified into four different genera: alpha, beta, gamma and delta coronaviruses [4,5,6]. SARS-CoV-2 has four structural genes that encode the spike (S), envelope (E), membrane (M), and nucleocapsid (N) proteins [7]. Amongst these structural proteins, the S and N proteins are often used to develop ELISA tests to detect antibodies specific for defined coronaviruses and also for SARS-CoV-2.

The incubation period of SARS-CoV-2 in humans ranges from 1 to 14 days. During this period, it is important to correctly identify infected patients, quarantine them and perform contact tracing to control transmission and spread [8,9]. Detection of SARS-CoV-2 RNA by real time RT-PCR in nasal, nasopharyngeal, and/or saliva swabs is currently the gold standard to diagnose SARS-CoV-2 infection in humans and other animals. However, the sensitivity of the RT-PCR greatly depends on the viral load, specimen type, the correct execution of the pharyngeal, nasal or oral swab, the timing of specimen collection regarding the onset of clinical symptoms, transportation of the sample and methods of analyses [10]. False positive results will put the patient in COVID-19 imposed restrictions such as quarantine at home, or into a COVID unit when showing respiratory signs. False negative RT-PCR tests can also occur and will contribute to transmission and spread of the SARS-CoV-2 agent. Therefore, the determination of, SARS-CoV-2-specific IgM antibodies after 3–6 days post infection (DPI) or IgG antibodies after 8 DPI can add significant value to identify SARS-CoV-2 infected people. SARS-CoV-2-specific antibodies can be measured by a variety of immunoassays that can provide detailed information on the antibody profiles of SARS-CoV-2-infected humans and animals [11,12].

ELISAs and virus neutralization tests are commonly used to investigate the antibody responses of humans to SARS-CoV-2. Amongst these, ELISA is a good option to measure antibody responses in both SARS-CoV-2-infected and -vaccinated people and animals in an economically affordable and high-throughput manner. In addition, measuring SARS-CoV-2 antibody levels in vaccinated people has prognostic value and offers information on protective immunity in vaccination trials and vaccine efficacy studies. There are several hundred immunoassays available worldwide to measure SARS-CoV-2 antibodies in humans [12,13]. However, the sensitivity, specificity, and accuracy of these assays varies significantly. The performance of these immunoassays mainly depends on the choice of the target antigen, on the nature, production and purification of the target antigen, the timing of the sample collection post infection/vaccination, and disparities in the selection of patient cohorts [13]. Therefore, ELISA tests need to be validated to accurately measure antibody responses to SARS-CoV-2 antigens in human sera. In this study, we developed three ELISA tests based on the recombinant spike (S), the subunit S1, and the receptor-binding domain (RBD) proteins of SARS-CoV-2. After establishment of these ELISAs, sera from RT-PCR-positive SARS-CoV-2-infected patients and from SARS-CoV-2-vaccinated humans in Istanbul, Turkey, were analyzed by in-house ELISAs and compared to commercial tests in order to determine the performance characteristics of the individual ELISA tests.

## 2. Materials and Methods

### 2.1. Study Population and Sampling

This study was performed on patients admitted to a private analysis laboratory and the Cerrahpasa Medical School of Istanbul University-Cerrahpasa, Turkey. The study population consisted of three groups. The first group was called “Infected”, the second group “Vaccinated”, and the third group “Before COVID-19”. Sera from the “Infected” and “Vaccinated” groups were collected between January 2021 and February 2021, after the SARS-CoV-2 vaccinations started in Turkey. Sera from the “Before COVID-19” group were collected before the detection of the first SARS-CoV-2 case in China (before December 2019); these samples were not expected to be positive for SARS-CoV-2 antibodies. The “Infected” group consisted of a total of 70 patients, the “Vaccinated” group consisted of 222 patients, and the “Before COVID-19” group consisted of 50 patients. The cohort age in each group was between 19 and 65 years. All infected individuals in the group “Infected” were clinically ill and were confirmed to be SARS-CoV-2-RNA positive by real time RT-PCR. The members of the “Vaccinated” group were vaccinated twice with an inactivated SARS-CoV-2 vaccine registered as CoronaVac, which was produced in China (Sinovac Life Sciences Co., Ltd., Beijing, China). The sera were collected 2 weeks after the booster vaccination.

### 2.2. Production of Recombinant Spike (S), S1 and Receptor Binding Domain (RBD) Proteins of SARS-CoV-2

The SARS-CoV-2 S, S1 and RBD proteins were produced as described previously [14,15,16]. Briefly, the expression cassettes containing SARS-CoV-2 S and S1 nucleotide sequences of BetaCov/Wuhan/WH04/2020 (Accession number: EPI-ISL-406801) were retrieved from the Global Initiative on Sharing All Influenza Data (GISAID) database. The alignments of S protein amino acids of BetaCov/Wuhan/WH04/2020, other SARS-CoV-2 isolates and other human coronaviruses (OC43, NL63, Huk-1 and 229-E) were performed in order to check similarities (Table 1). Nucleotide sequences were codon-optimized for expression in human embryonic kidney (HEK) 293T cells. The N-terminal signal sequence of the S protein was replaced with the signal sequence of the human CD33 myeloid cell surface antigen, and the C-terminus was fused with the T4 foldon sequence (GSGYIPEAPRDGQAYVRKDGEWVLLSTFL) and the CaptureSelect C-tag sequence (EPEA) for affinity purification. The protein expression cassettes were commercially synthesized (GeneArt, ThermoFisher Scientific, Regensburg, Germany) and cloned into the pcDNA3.1 expression vector. The nucleotide sequence encoding the spike RBD protein of the SARS-CoV-2 isolate Wuhan-Hu-1 (GenBank accession: MT380725.1) plus two strep-tags (IBA Lifesciences, Germany) on the 3′ end were used and cloned into the mammalian expression vector pHL (Addgene, Watertown, MA, USA) [16].

The recombinant plasmids were transfected into HEK-293T cells using Lipofectamine LTX (ThermoFisher Scientific, 15338100) according to the producer’s protocol. Transfected HEK-293T cells were cultured in DMEM (Thermo Fisher Scientific, Chicago, IL, USA). Supernatants from transfected cells were harvested on day 3 post-transfection and were centrifuged at 4000× *g* for 20 min. The S and S1 proteins were purified by affinity chromato-graphy CaptureSelect™ C-tag Affinity Matrix (Thermo Fisher Scientific). The RBD protein was purified using Strep-Tactin^®^ (IBA Lifesciences, Germany). After purification, proteins were dialyzed in phosphate-buffered saline (PBS; Sigma (St. Louis, MO, USA)) overnight. The concentrations of purified recombinant S, S1 and RBD proteins were determined by Pierce^TM^ BCA Protein Assay Kit (Thermo Fisher Scientific), and the purity was assessed by sodium dodecyl sulphate–polyacrylamide gel electrophoresis (SDS-PAGE).

### 2.3. Validation of S, S1 and RBD ELISAs with Human Sera Analyzed by Commercial Test Kits

The in-house indirect ELISA (iELISA) protocols using S, S1 and RBD proteins were validated as described previously [16,17]. The validation studies were performed as follows: Initially, a total of 292 sera from 222 SARS-CoV-2-vaccinated and 70 SARS-CoV-2-infected humans (clinically ill and confirmed SARS-CoV-2-RNA positive by RT-PCR) were tested using a commercial ELISA (SARS-CoV-2 IgG II Quant, Abbott Diagnostics, IL, USA) for the presence of SARS-CoV-2 IgG antibodies. The SARS-CoV-2 IgG II Quant assay is a microparticle immunoassay used for the quantitative and qualitative determination of IgG antibodies specific for the RBD of the spike protein of SARS-CoV-2 in human sera. This kit was licensed in Turkey and is widely used in research and diagnostic institutes. The results of the commercial ELISA were used to calculate the specificity and sensitivity of the in-house iELISAs. Next, the 292 sera were analyzed by the in-house iELISA methods. For this purpose, 96-well ELISA plates (Nunc MaxiSorb, Thermo Fisher Scientific) were coated with 100 ng of either S, S1 or RBD proteins per well in 50 μL PBS buffer (Sigma, C-3041) overnight at 4 °C. The next morning, plates were washed 3 times, and non-specific interactions blocked for 1 h at room temperature with a blocking buffer (PBS containing 3% [*w*/*v*] skim milk powder and 0.1% Tween 20). This was followed by another wash step (3×), and then 100 μL of test sera, diluted 1:50 in PBS-Tween plus 1% (*w*/*v*) skimmed milk non-fat powder (BioShop-Canada), was added and incubated for 2 h at room temperature. Each serum sample was tested in duplicate, and each test plate included duplicate negative human sera. After another wash step, HRP-conjugated goat anti-human antibody (Santa Cruz Biotechnology, Cat. No. sc2428, Dallas, TX, USA), diluted 1:3000 in blocking buffer, was added to the wells and incubated at room temperature for 1 h. Then, 100 μL of TMB substrate (ThermoScientific-C34021) solution was added to each well and incubated at room temperature for 10 min. The reaction was stopped by adding 50 μL/well of 2 M H_2_SO_4_, and absorbance (optical density) was measured at 450 nm using a microplate reader (SLT-Spectra, SLT Lab instruments, Germany).

### 2.4. Surrogate Virus Neutralization Test (sVNT)

In order to detect neutralizing activity against SARS-CoV-2, a commercial (Euroimmun NeutraLISA, Germany) SARS-CoV-2 Surrogate Virus Neutralization Test Kit was used. This test detects RBD-specific antibodies blocking the RBD-angiotensin converting enzyme 2 (ACE2) receptor interaction in this ELISA system. In this study, all human sera found positive by the in-house S, S1, RBD-ELISAs and the commercial ELISA kit were tested by the surrogate virus neutralization test as described by the manufacturer (Euroimmun NeutraLISA, Germany).

### 2.5. Calculation of Performance Characteristics

Specificity, sensitivity, and cut-off values were calculated by using the receiver operating characteristic (ROC) curve for the S-, S1- and RBD-based in-house ELISA methods. The kappa value and the area under the curve (AUC) were used to determine the agreement of results between the different ELISA methods and the results from the sVNT [18]. All statistical analyses were performed using the MedCalc (Version 20.010) software.

## 3. Results

### 3.1. Assessment of S, S1 and RBD-Specific iELISAs Developed in this Study

**Figure 1:**
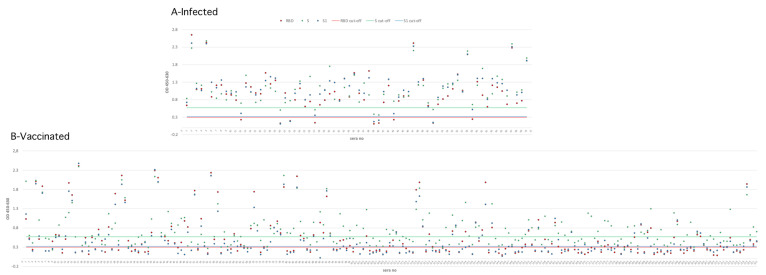
OD values of sera analyzed by the in-house S, S1 and RBD ELISAs. (**A**) Human sera infected with SARS-CoV-2; (**B**) Human sera vaccinated with SARS-CoV-2 inactivated vaccine.

The SARS-CoV-2-specific, S-, S1- and RBD-based iELISAs, developed in-house in this study, were used to detect the presence of SARS-CoV-2-specific antibodies in serum collected from three groups of patients; also, a commercial SARS-CoV-2 antibody ELISA test was used. The OD cut-off values for the in-house iELISAs were set at an OD of 0.570, 0.320 and 0.300 for the S-, S1- and RBD-based iELISAs, respectively (Figure 1). Of the 70 sera from SARS-CoV-2-infected patients, 92.8% (65/70) tested positive with the in-house S iELISA, 92.8% (65/70) with the in-house S1 iELISA, 87.14% (61/70) with the in-house RBD iELISA, and 95.7% (67/70) with the commercial ELISA test, while 90% (63/70) were positive in the sVNT (Table 2). Of the 222 sera from SARS-CoV-2-vaccinated patients, 54.5% (121/222) tested positive with the in-house S iELISA, 47.7% (106/222) with the in-house S1 iELISA, 52.2% (116/222) with the in-house RBD iELISA, and 62.6% (139/222) with the commercial ELISA test, while 49.5% (110/222) were positive in the sVNT (Table 2).

Fifty archival sera (group “Before COVID-19”), obtained before the first COVID-19 patient was reported in Turkey, were analyzed for the presence of SARS-CoV-2-specific antibodies by the in-house iELISAs. One human serum sample was found to be positive for SARS-CoV-2 antibodies by both, the in-house S- and S1-based iELISAs. The OD values of the positive serum sample ranged between 0.850–0.973 in the in-house S and S1 iELISAs. This serum sample tested negative with the in-house RBD iELISA and the commercial ELISA kit.

### 3.2. Performance Characteristics

The sensitivity of the in-house S-, S1- and RBD-based iELISAs was found to be 88.44, 90.17 and 95.38%, while the specificity was 72.27, 89.08 and 89.92%, respectively. The area under the curve (AUC) values were 0.777 for the in-house S iELISA, 0.926 for the S1 iELISA, and 0.959 for the RBD iELISA (Table 3).

When the results of all three in-house iELISAs were analyzed by kappa statistics, the kappa values were determined to be 0.62, 0.79 and 0.86 for the S-, S1- and RBD-based iELISAs, respectively (Table 3). The kappa values indicated that the S, S1 and RBD iELISAs had “good” to “excellent” agreement based on established criteria [18]. The kappa value (0.86) of the in-house RBD-based iELISA was in excellent agreement (Table 3).

### 3.3. Surrogate Virus Neutralization Test (sVNT)

Sixty-three (90%) out of 70 sera from SARS-CoV-2-infected patients tested positive in the sVNT, while 110 (49.54%) out of 222 sera from vaccinated humans were found to be positive in the sVNT (Table 2). A comparison of the number of positives in vaccinated patient sera obtained from iELISAs and the sVN test is shown in Table 4. Ninety (90) of S, 94 of S1 and 104 of RBD positive sera were also positive in the sVN test. When sera from the “Before COVID-19” group were assessed using surrogate virus neutralization tests, they were all negative, including the one positive by the S and S1 iELISAs.

## 4. Discussion

Public health authorities around the world are trying to mitigate the SARS-CoV-2 (COVID-19) pandemic, especially since widespread infections with the Omicron variant of concern (VOC) seem to cause vaccine failures and diagnostic problems [7,19,20,21]. Turkey is geographically located between Europe and Asia as a bridge between these two continents. To control emerging SARS-CoV-2 VOCs, it is important to limit their spread between continents. The increase in human mobility due to increased globalization, trade, and refugee migrations at the borders of Middle East countries creates significant challenges. Strict border regulations, early diagnosis, early warning, biosecurity, isolation-quarantine and vaccination are used to prevent and control pandemics such as SARS-CoV-2. Therefore, in this study, we developed and evaluated 3 in-house ELISAs by using the recombinant S, S1 and RBD proteins of SARS-CoV-2 as diagnostic targets to determine antibody responses to vaccination and to natural SARS-CoV-2 infections in humans.

Sensitivity, specificity, AUC and kappa values are important performance characteristics of diagnostic tests; molecular and serological assays are mainly used for the diagnosis of SARS-CoV-2 and determination of RNA and antibody levels in humans, respectively. Detection of SARS-CoV-2 RNA by RT-PCR in nasal and nasopharyngeal swabs is currently used for molecular diagnostic purposes. Serological tests, particularly ELISAs, are used for the determination of antibody levels in both infected and vaccinated people to aid in diagnosis. The use of RT-PCR and serological tests for SARS-CoV-2 diagnosis varies according to the patient’s infection status (onset or long term) and disease severity (with or without symptoms) [10]. Both tests can be combined and used for diagnosis, but this is not economical [22]. However, having results from both tests will help in diagnosing a SARS-CoV-2 infection with higher confidence. The sensitivity of RT-PCR was found to be between 50–95% depending on many factors such as sampling time, conditions of transport, and how the test is performed [10,12,23].

For this reason, suspect COVID-19 patients could be SARS-CoV-2 positive (i.e., shedding virus) or negative (not shedding virus, or suffering from a different respiratory disease). In the latter cases, the use of an antibody test such as an ELISA is recommended. For these cases, it has been found that if only RT-PCR is used for diagnosis, the rate of detection of true positives is 51.6%, while the detection rate increases to 98.6% when ELISA tests detecting SARS-CoV-2-specific IgM and IgG antibodies are included [23]. Since ELISA tests are needed for SARS-CoV-2 diagnosis and for determining antibody levels in a population of naïve, infected and vaccinated people, we developed three in-house ELISAs using the recombinant S, S1 and RBD proteins of SARS-CoV-2.

The N, S, S1 and RBD proteins of SARS-CoV-2 are commonly used as target antigens in serological tests [13,24,25,26,27,28]. It has been reported that recombinant S protein-based ELISA kits are better in detecting SARS-CoV-2-specific antibodies than N protein-based ELISA kits [24,26,27,29,30,31]. The S protein induces neutralizing antibodies [22] mainly directed against the RBD portion of the S protein. Therefore, the level of antibodies against RBD correlates with the level of protective antibodies against SARS-CoV-2. In the study by Zhang and others [32], the highest IgM and IgG values were obtained by tests with the recombinant S1 and RBD proteins. In another study, an S-based ELISA was found to be more sensitive than an N-based ELISA [26]. This may be due to an earlier expression of S antigens versus N antigens in infected individuals. Therefore, testing for S-, S1- or RBD-specific antibodies by ELISA is recommended in suspect COVID-19 cases that are RT-PCR negative [26]. It should be noted that later, after infection (>14 days), an N-based ELISA could also be used [23]. In this study, for the reasons explained above, three in-house ELISA tests were developed using the recombinant SARS-CoV-2 S, S1 and RBD proteins produced in HEK cells.

During SARS-CoV-2 infections in humans, SARS-CoV-2-specific antibodies can be detected 5–6 days after virus infection, and the detection time varies according to the antibody type [33]. IgMs can be detected 6–7 days (minimum 5, maximum 10 days) and IgGs 10–12 days (minimum 7, maximum 14 days) after the first disease symptoms are observed [23,26,28]. Therefore, the sampling time affects the results and performance of both, molecular and serological tests. Because of this, in the present study, serum of both vaccinated (inactivated vaccine; CoronaVac) and SARS-CoV-2-infected individuals were examined with in-house ELISA tests, and sera were taken from patients 14 days after vaccination and during the clinical illness as confirmed by RT-PCR.

Various studies have shown that analyses of RT-PCR-negative cases by ELISA for IgM or IgG antibodies helps in the diagnosis of COVID-19 [23,27,33,34]. According to the results of RT-PCR and serological studies performed with swabs and blood taken from infected people within 10–14 days post infection, the sensitivity of RT-PCR tests decreased, while the sensitivity of serological tests increased at later time points post infection [35]. A meta-analysis of 599 studies indicated seroconversion at days 7, 14, 21, and 28 and thereafter was 37.5%, 73.3%, 81.3%, and 72.3% for IgM and 73.3%, 35.4%, 80.6% and 93.3% for IgG, respectively. By day 21, the IgM sensitivity was 87.2%, and the specificity was 97.3%, while the IgG sensitivity was 91.3%, and the specificity was 96%. It was emphasized that blood taken on day 14 post infection is important for the accuracy of the ELISA test in terms of detecting IgG [36]. Therefore, in this study, sera were collected 14 days after vaccination.

Different sensitivity and specificity values for detecting SARS-CoV-2-specific IgM and IgG antibodies have been reported previously [28,37,38]. In one study, the sensitivity for detection of IgM antibodies in COVID-19 patients was 48%, and of IgG antibodies approximately 89% [38]. In another study, the sensitivity was found to be 72% for IgM and 100% for IgG antibodies [28], whereas the specificity of the ELISA has been reported as 100% for IgM and 91% for IgG [38]. In another study, specificity was found to be 98.7% for IgM and 100% for IgG [28]. Results of a meta-analysis of 5016 studies revealed that the sensitivity for ELISAs was between 75.6–90.9%, while the specificity was 86.6%–99.7% [37]. Comparing the results of the aforementioned analysis with the results of the present study in terms of sensitivity and specificity reveals the following: the sensitivity of the S, S1 and RBD ELISAs in this study was 88.44%, 90.17%, and 95.38%, respectively, while the specificity was 72.27%, 89.08%, and 89.92%, respectively. According to these results, the RBD-, S1, and S-based ELISA tests had acceptable sensitivity values, whereas the RBD and S1 ELISA tests had acceptable specificity values. We conclude that the in-house RBD-based ELISA is the best for use in diagnosis and the determination of SARS-CoV-2 antibody levels in both infected and vaccinated individuals.

The Wantai ELISA (http://www.ystwt.cn/covid-19/, 1 June 2022), a commercially available kit, is based on the S1 and RBD antigens. The S1 antigen is also used in the ELISA developed by Euroimmun (https://www.fda.gov/media/152747/). In one study, it was reported that the Wantai ELISA kit was better in measuring SARS-CoV-2-specific IgG and IgA antibodies than the Euroimmun ELISA kit [34]. In addition, it was reported that an N-based ELISA developed by ID-Vet (ID Screen^®^ SARS-CoV-2 Double Antigen Multi-species ELISA-IDvet (https://www.innovative-diagnostics.com/, 5 September 2022) is more specific than the S1 ELISA from Euroimmun [39]. A correlation was found between S1-specific antibody levels and neutralizing antibodies (tested by microneutralization) 3–4 weeks after infection. However, the same correlation was not found with N-specific antibody levels [40]. The specificities of the Wantai, Euroimmun, Abbott (https://www.abbott.com) and Roche (https://diagnostics.roche.com/global/en/home.html) ELISA kits are approximately 99% but their respective sensitivities vary [22,35]; it is recommended that the tests be performed 10 days after the appearance of first symptoms. In a study performed by Spicuzza and others [41], the sensitivity of antibody tests was found to be 83%, and the specificity 93%. In another study, the sensitivity for IgM antibodies was 48%, and the specificity was 100%, while for IgG antibodies, the sensitivity was 88.9%, and the specificity was 90.9% [25]. In comparisons of data obtained in the present study with the Abbott kit, the kappa values were found to be 0.62, 0.79 and 0.86 for the in-house S, S1 and RBD ELISAs, respectively; therefore, it was determined that there was “good agreement” for the S and S1 ELISAs and “excellent agreement” for the RBD ELISA. These results indicate that all in-house ELISAs developed in this study, and especially the RBD ELISA, had excellent agreement based on established criteria reported previously [18]. The sensitivities of the in-house ELISA tests were 88.44%, 90.17% and 95.38% for the S, S1 and RBD ELISAs, respectively, and their specificities were 72.27%, 89.08% and 89.92%, respectively. These values are similar to the standards recommended by the FDA and EUA, namely that the sensitivity should be around 90% and the specificity should be around 95% [42].

Interestingly, the reaction of one of the 50 archival human sera taken before the COVID-19 pandemic was positive with the S and S1 SARS-CoV-2 antigens in the in-house ELISAs in the present study. One possible explanation is that the sera cross-reacted with these antigens due to infection with other human coronaviruses such as the 229E, OC43, HKU1 or NL63 strains [24,43]. In another ELISA study based on the S protein, a low level of cross-reactivity was detected with MERs CoV and SARS-CoV, while no cross-reactivity was detected with the 229E, OC43, HKU1 or NL63 strains [27]. No cross-reactivity was detected in one ELISA study based on the N protein [23], while cross-reaction was found in another study [31]. In a study conducted with commercial ELISA kits, cross-reactivity was found with OC43 in one kit and two sera, whereas no cross-reactivity was found with other human coronaviruses, and the specificity of the IgG ELISA was reported as 91.9% [24]. The positive reaction in the in-house S and S1 ELISAs with one of the 50 archival human sera samples seen in this study might indicate cross-reactivity with other human coronaviruses. Therefore, the RBD iELISA seems to be the best test system for use of diagnosing COVID-19 infection and vaccine response in people.

Since we do not have BSL-3 facilities at our institution, the ELISA-positive sera could not be tested by a classical virus neutralization assay. Therefore, we analyzed human sera found to be positive in the in-house S, S1, and RBD ELISAs and the commercial ELISA kit using a surrogate virus neutralization test. The results revealed that 63 of 70 SARS-CoV-2-infected human sera and 110 of 222 SARS-CoV-2-vaccinated human sera were sVNT positive. These results indicate that the majority of infected humans had neutralizing antibodies to SARS-CoV-2. In contrast, only approximately 50% of the vaccinated humans were positive by sVNT. This could be due to the low capacity of a killed vaccine formulation (CoronoVac) to produce high titers of neutralizing antibodies or the possibility that 14 days post booster vaccination was not long enough to achieve a good neutralizing antibody response that could be detected by the sVNT.

## 5. Conclusions

There is a need for a validated, highly sensitive, highly specific and accurate serological test that can be used in the diagnosis of COVID-19 and help in the determination of the immunity level in a specific community or country/region. Such a test will aid in diagnosing COVID-19 infections and eventually mitigate transmission and the spread of infection. In this study, the sensitivity and specificity of the indirect ELISA test developed using the SARS-CoV-2 RBD protein produced in HEK cells were high and fulfill international standards. Using the in-house RBD ELISA, public health authorities will be able to determine the immunity levels of selected communities and determine their vaccination status; this will aid in defining vaccination strategies. Ultimately, it will contribute to mitigation of the SARS-CoV-2 pandemic.

## Figures and Tables

**Table 1:**
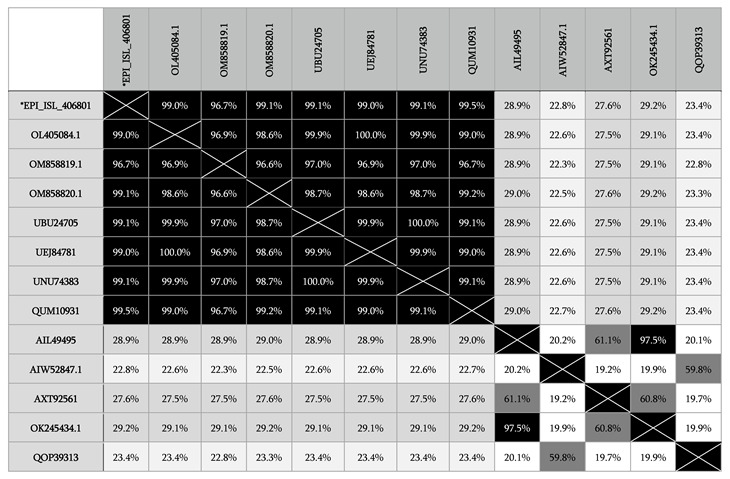
Amino acid alignment of SARS-CoV-2, BetaCov/Wuhan/WH04/2020 and other human coronaviruses (OC43, NL63, HKU1, 229E) for similarity. *EPI-ISL406801: The S sequences of “BetaCov/Wuhan/WH04/2020” were used in this study. OK245434.1: Human coronavirus OC43; OL405084.1: Human coronavirus SARS-CoV-2; OM858819.1: Human coronavirus SARS-CoV-2-Delta variant; OM858820.1: Human coronavirus SARS-CoV-2-Omicron variant; QOP39313: Human coronavirus 229E; QUM10931: Human coronavirus SARS-CoV-2; UBU24705: Human coronavirus SARS-CoV-2; UEJ84781: Human coronavirus SARS-CoV-2; UNU74383: Human coronavirus SARS-CoV-2; AIL49495: Human coronavirus OC43; AIW52847: Human coronavirus NL63; AXT92561: Human coronavirus HKU1.

**Table 2:** Number of positive and negative sera from SARS-CoV-2-infected or -vaccinated patients analyzed by in-house S-, S1- and RBD-based iELISAs, a commercial ELISA and the sVNT test.

Human Sera	In-House S iELISA		In-House S1 iELISA		In-House RBD iELISA		Commercial ELISA Kit (Abbott)		Surrogate Virus Neutralization Test	
	*Pos*	*Neg*	*Pos*	*Neg*	*Pos*	*Neg*	*Pos*	*Neg*	*Pos*	*Neg*
Infected	65	5	65	5	61	9	67	3	63	7
Vaccinated	121	101	106	116	116	106	139	83	110	112
Total	186	106	171	121	177	115	206	86	173	119

Pos = Positive; Neg = Negative.

**Table 3:** Performance characteristics of the in-house S, S1 and RBD iELISAs.

Performance Characteristics	In-House S iELISA	In-House S1 iELISA	In-House RBD iELISA
Sensitivity	88.44%	90.17%	95.38%
Specificity	72.27%	89.08%	89.92%
Cut-off value	0.570	0.320	0.300
AUC	0.777	0.926	0.959
Kappa value	0.62	0.79	0.86

AUC = Area under the curve.

**Table 4:** Comparison of number of positives in SARS-CoV-2-vaccinated patient sera obtained from iELISAs and sVN tests. 1: Number of sera positive in both S iELISA and sVN tests. 2: Number of sera positive in both S1 iELISA and sVN tests. 3: Number of sera positive in both RBD iELISA and sVN tests. 4: Number of sera positive in both S and S1 iELISA. 5: Number of sera positive in both S and RBD iELISA. 6: Number of sera positive in both S1 and RBD iELISA.

Test No.	Tests	Number of Positives-(%)
1	S iELISA and sVN	90 (40.5)
2	S1 iELISA and sVN	94 (42.3)
3	RBD iELISA and sVN	104 (46.8)
4	S and S1 iELISA	91 (40.8)
5	S and RBD iELISA	101 (45.4)
6	S1 and RBD iELISA	98 (44.1)

## Data Availability

Data of this study are available from the corresponding author upon request.
